# Mebendazole Compared with Secnidazole in the Treatment of Adult Giardiasis: A Randomised, No-Inferiority, Open Clinical Trial

**DOI:** 10.1155/2011/636857

**Published:** 2011-11-20

**Authors:** Pedro Almirall, Angel A. Escobedo, Idalia Ayala, Maydel Alfonso, Yohana Salazar, Roberto Cañete, Sergio Cimerman, Martha Galloso, Ilmaems Olivero, Maytee Robaina, Karen Tornés

**Affiliations:** ^1^Faculty of Medicine “Manuel Fajardo”, 10400 Havana City, Cuba; ^2^Academic Paediatric Hospital “Pedro Borrás”, 10400 Havana City, Cuba; ^3^Carlos J. Finlay Hospital, 11500 Havana City, Cuba; ^4^Cuban Institute of Gastroenterology, 10400 Havana City, Cuba; ^5^National Institute of Hygiene, Epidemiology and Microbiology, 10300 Havana City, Cuba; ^6^Institute of Infectology “Emilio Ribas”, 04610-002 São Paulo, SP, Brazil; ^7^National Centre Coordinator of Clinical Trials, 11600 Havana City, Cuba

## Abstract

To compare the efficacy and safety of mebendazole and secnidazole in the treatment of giardiasis in adult patients, a single-centre, parallel group, open-label, randomized non-inferiority trial was carried out. One-hundred and 26 participants who had symptomatic *Giardia* mono-infection took part in the study. Direct wet mount and/or Ritchie concentration techniques and physical examinations were conducted at the time of enrolment and at the follow-up visit. The primary outcome measure was parasitological cure, performed at 3, 5, 10 days post-treatment. Negative faecal specimens for *Giardia* were ensured by the same parasitological techniques. At follow up (day 10) the parasitological cure rate for the *per protocol* populations was 88.7% (55/62) for MBZ and 91.8% (56/61) for SNZ. For the intention to treat populations the cure rate at the end of treatment was 85.9% (55/64) for MBZ and 90.3% (56/62) for SNZ. Both analyzes showed there was not significant statistical difference between MBZ and SNZ treatment efficacy. Both drugs were well tolerated, only mild, transient and self-limited side effects were reported and did not require discontinuation of treatment. A 3-day course of mebendazole seems to be as efficacious and safe for treatment of giardiasis as a single dose of secnidazole in adults.

## 1. Introduction

*Giardia lamblia*, the causative agent of giardiasis, is one of the commonest intestinal parasitic protozoan infections diagnosed world-wide. The spectrum of this infection may range from asymptomatic shedding of giardial cysts to symptomatic giardiasis, being responsible for abdominal cramps, nausea, acute or chronic diarrhoea, with malabsorption, and failure of children to thrive [1]. 

 Five nitroimidazole compounds are considered to be the first-line regular therapy for this infectious disease. However, whilst their therapeutic benefits are generally accepted, treatment failures are often reported [2, 3], and that is why a variety of new approaches to the treatment of giardiasis have been entering into clinical practice [4]. 

 Evidence from uncontrolled case series and clinical trials in paediatric patients suggests that mebendazole (MBZ) might have a role in the treatment in this parasitosis and that its therapeutic effect is achieved without an accompanying increase of side effects [5–8]. Despite the number of articles published concerning the use of this drug in paediatric patients with giardiasis, the information about the use of MBZ in adult giardiasis is scarce. 

 The aim of the study was to determine whether MBZ is as efficacious and safe as secnidazole (SNZ), a 5-nitroimidazole with a high rate of healing and low cost, in the treatment of adult patients with giardiasis.

## 2. Subjects and Methods

### 2.1. Study Design and Patients

A single-centre, randomised, unblinded, parallel-group, open-labeled, no-inferiority clinical trial was carried out at “Carlos J. Finlay” Hospital in Havana City, Cuba. The study protocol was reviewed and approved by the institutional review board of the hospital.

 The study subjects were adult patients (17 years old or older) who had been referred by general practitioners or who presented at the hospital seeking treatment for symptomatic, acute *G. lamblia* monoinfection (proven by microscopical examination of faecal samples as direct wet mounts and/or after Ritchie concentration). Patients were not allowed to participate if they had previously received any antiparasitic drug within 1 month before entering the study. Other exclusion criteria had known hypersensitivity to any of the drugs in use and suspected immunodeficiency, had hepatic, renal, cardiovascular, or haematological disease, and had concomitant use of other drugs. Women were ineligible if they were pregnant or lactating. Women of childbearing potential were only admitted if they were using safe, adequate, and medically accepted contraceptive precautions. Patients fulfilling the inclusion criteria received written and oral information on the aims of the study before asking for their participation decision. Enrolment was also dependent on the production of an informed consent form signed. The specific objectives were to determine the parasitological answer of the patients once they took MBZ or SNZ and to identify and evaluate the intensity of the possible adverse events once patients took MBZ or SNZ.

 It was considered that the experimental treatment with MBZ was not inferior to the treatment with SNZ if the proportion of the patients cured parasitologically with MBZ was 20% less than the proportion of the patients cured with the therapeutic with SNZ. The sample size was estimated for the two treatment groups (*n*), based on the assumption: a response proportion of 85% for both groups of treatment, with two sides, *α* level = .05 and a power of 0.9. This indicated that 110 subjects would be needed (i.e., 61 in each treatment arm) and 126 were enrolled. All of them were randomly assigned to one of the two parallel groups. A randomized list of two indifferent blocks was generated automatically by a computer to assign the patients to one or another group of treatment. Medical doctors whose assisted the patients carried out the assignation according to the list conformed to receive either MBZ (200 mg three times daily for 3 days, according with earlier trials in which 200 mg three times daily for 3 days produced 78% parasitological cure rates in children) [7] or SNZ (2 g as a single dose).

 Clinical signs and symptoms were recorded in a written evaluation form for each patient at the beginning of the study period through the interrogatory in the place of the consultation. Adverse events, defined as signs and symptoms that first occurred or became more severe following the treatment, were recorded using a standardized questionnaire, taking into account its intensity and duration. The events are classified as mild: occasional event without normal activities interferences; moderate: events where there are normal activities interferences, but occasional; serious: those adverse events that required hospitalisation, were life threatening, or resulted in a persistent or significant disability or death. 

### 2.2. Followup

The efficacy of the chemotherapy was assessed by the microscopical examination (as direct wet mount and Ritchie concentration) of faecal samples collected soon (3, 5, and 10 days) after treatment completion in order to avoid the bias that would be introduced by reinfection. Patients and physicians knew the treatment assignment; nevertheless, the laboratory personnel who analysed the faecal samples to determine the parasitological outcome were blind to patient's treatment assignment. The patient was only considered to be cured if no *Giardia *cysts or trophozoites could be found in any of the three posttreatment faecal samples. Patients were evaluated again to ask about their symptoms and signs once they had the results of their faecal samples after the treatment.

 Criteria for patient withdrawal from the study included (a) the patient's desire to withdraw from the study; (b) violation of the study protocol; (c) onset of a serious medical condition.

### 2.3. Statistical Analysis

 Baseline characteristics and adverse events were compared using *χ* ^2^ tests in categorical data, for continuous data Student's *t*-test was used.

The hypothesis test for proportion equivalence and the associated 2-sided 95% CI for the difference was estimated to evaluate the equivalence of the principal variable “parasitological efficacy” and it was carried on by intention to treat as if as per protocol [9, 10]. The no-inferiority margin defined in the primary analysis was based on absolute cure rate differences. No inferiority of MBZ over SNZ was accepted [in a two-side 0.05 level test] if the upper bound of the 95% CI around the estimated difference in parasitological cure rates lies below 20%.

## 3. Results

### 3.1. Patient Characteristics

**Figure 1:**
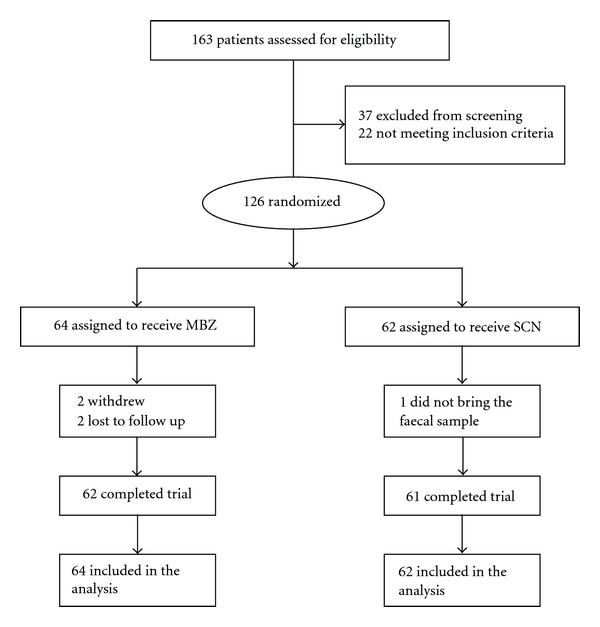
Flow diagram of the clinical trial progress.

From March 2005 through February, 2006 a total of 163 patients presenting to the trial site were screened and 126 were eligible and agreed to be enrolled; 123 of them successfully completed the study (Figure 1). In the research, the efficacy and safety analysis was done *per protocol* as well as by intention to treat. Baseline demographic characteristics are summarised in Table 1. Overall, there was a slightly higher proportion of males compared to females entering the study (69% versus 30.9%); however, there were no significant differences between the groups concerning gender distribution neither in terms of age (*P* > 0.05). Concerning clinical features, nausea was the only one that was more reported by patients who would receive SNZ and had statistically significant difference (*P* < 0.05). 

### 3.2. Efficacy Assessment

Table 2 displays the efficacy results. No inferiority was found for both kinds of analysis, *per protocol* and *intention to treat*. At followup, parasitological cure showed by *per-protocol* analysis was experienced by 88.7% (55/62) and 91.8% (56/61) of the patients treated in the MBZ and SNZ groups, respectively. This gave an absolute difference of 3.1% (two-side 95% IC −1; 0.12); and *P* value associated of 0.0008. When were included all randomized patients in *intention-to-treat* analysis, the cure rates at the end of treatment were 85.9% (55/64) for MBZ and 90.3% (56/62) for SNZ, the two-sided 95% IC −1; 0.14; *P* = 0.003. Both analyses show the upper limit IC for the population proportion difference was lesser than 0.2, the difference chosen. 

### 3.3. Safety Assessment

No patients in any treatment group discontinued the study due to adverse events. Adverse events during treatment are shown in Table 2. Both drugs were well tolerated; only mild, transient, and self-limited adverse events were reported; most of them developed between 30 minutes and six hours after treatment but had subsided within 24 hours. There was no statistically significant difference between the total numbers of patients who experienced an adverse event in the two treatment groups. The event most commonly reported in MBZ treatment group was abdominal pain [12/64, (18.7%)] versus 22.5% in the SNZ treatment group. Apart from bitter taste and dizziness, which were more frequently reported amongst those who took SNZ and had statistically significant differences, there were no statistically significant differences in the report of any of the other adverse events reported. 

## 4. Discussion

The present study gives additional information about the use of MBZ in the treatment of adult patients with giardiasis. Despite initial studies carried out by Hutchison et al. in 1975 [11] which had demonstrated the effectiveness of MBZ in the treatment of *Giardia* infection, the full potential of this alternative regimen was not immediately apparent. It could be due, in part, because there has been some debate concerning to the efficacy of this drug in this indication; while Al-Waili and Hasan [12] reported a high *Giardia* eradication with this drug, Gascon et al. [13] and di Martino et al. [14] failed to clear parasitic infection or symptoms in their patients. *In vitro* studies in which it has been demonstrated that MBZ at low concentrations (0.05 micrograms/mL) has a static effect on *G. lamblia* growth and has a lethal activity at a concentration fivefold lower (0.3 micrograms/mL) than that necessary for metronidazole have also led to the present situation where MBZ is recognized to play an important role in the treatment of giardiasis [15].

 In paediatric practice, MBZ has been used as an efficacious and safe treatment option. A number of studies have been performed in children comparing this drug with some of the currently available antigiardial drugs. An overall analysis of the results shows that MBZ possesses an efficacy which is comparable to secnidazole 30 mg as a single dose (78.1% versus 79.4%) [7], to that of a 7-day course of metronidazole (86% versus 90%) [8], and to that achieved with 3-day course of nitazoxanide (71% versus 75%) and also equivalent to 5-day course of quinacrine (78.7% versus 83.6%) [5]. However, the efficacy of 600 mg of MBZ divided into three doses, in a single day, was significantly lower than 50 mg/kg of tinidazole taken as a single dose (63.9% versus 81.9%) [6]. Nevertheless, these studies have not only confirmed the potency and safety of MBZ, but have also served to further clarify the clinical activity of benzimidazole carbamates as antiprotozoal agents. 

 One of the purposes of our study was to compare the efficacy of MBZ with SNZ in adult patients with giardiasis, using as criteria the absence of trophozoites or cysts from treated patients. It has been demonstrated that MBZ is a suitable candidate to treat giardial infections in adult patients as equivalent SNZ. While the little efficacy difference in favour of SNZ in terms of parasitological cure rates was unsurprising, it was interesting to note that there was no statistically significant difference between the groups. Also, when adverse events in general were evaluated, both treatments were well tolerated with similar adverse event profiles; however, there was an advantage with MBZ. This drug was well tolerated as well as efficacious. In no case did side effects lead to discontinuation of the treatment. The most frequently reported adverse event was abdominal pain, which was not unexpected taking into account previous articles reported in children [5–8]. These findings are consistent with clinical experience with MBZ. Even in studies when this drug has been used in higher doses, in divided doses after fat-rich meals, and for the treatment of the hydatid disease, there is little evidence of systemic effects, suggesting that MBZ has a wide margin between its antigiardial therapeutic effects and its adverse events [16], which seems to be confirmed by Rippmann et al. [17] and Franchi et al. [18]. Studies have shown that MBZ is relatively poorly absorbed from gastrointestinal tract [19].

 While SNZ offers the advantage of single-dose therapy and higher rate of efficacy, MBZ has also the advantage of less frequent dosing than metronidazole, other 5-nitroimidazole very frequently used, and shorter duration of therapy and, at the same time, is better tolerated, factors associated with improved treatment compliance. The simplicity of the SNZ treatment must be placed in one side of the balance, resting in the other side adverse events as bitter taste which was significantly reported in this group of treatment and is related with this and other 5-nitroimidazolic drugs and the possibility of therapeutic failures. 

 According to the results obtained, MBZ appears to be also an important option for the treatment of *G. lamblia* infections in adults, too. Together, with the beneficial therapeutic effect, several other characteristics of MBZ may enhance its potential to giardiasis therapy, first, for patients intolerant to 5-nitroimidazole compounds. Second, the use of this drug has lower incidence of mild and self-limited adverse events, may be due to its poor absorption from the gastrointestinal tract, the lack of interference with the balance of the microbial ecosystem of the gut, and the possibility of clearing or reduceing the parasitic burden of some of the common intestinal helminths which may co-occur, reducing at the same time the environment contamination with eggs of other sensitive organisms, for example, intestinal nematodes, which are especially frequent in tropical climates throughout the world. All of these pinpoint MBZ as a wise treatment option in multiple clinical settings. 

 We consider that MBZ has its role in the antigiardial armamentarium and should be considered as an alternative, especially when first-line drugs have failed, were not tolerated, or are not available. Also, MBZ could be possibly taken as adjunctive therapy in combination with other available antigiardial drugs targeting different pathways in order to offer potentially higher cure rates. This seems to be a promising task and would provide a focus for future studies. It is also important that the good clinician strives to achieve an overview of the beneficial effects of this treatment option in a given patient, taking into account all of the various drug effects for which a patient would be benefited and put it into a balance with the other drugs currently in use for giardiasis.

## Figures and Tables

**Table 1:** Baseline characteristics of the patients.

	MBZ		SNZ	
Characteristic	*n* = 64		*n* = 62	
	No.	(%)	No.	(%)
Gender				
Male	45	(70.3)	42	(67.7)
Female	19	(29.7)	20	(32.5)
Age (y)				
Median	31.9		38.1	
Range	19–62		17–59	
SD	±12.9		±13.4	
Symptoms				
Abdominal pain	42	(65.6)	47	(75.8)
Diarrhoea	13	(20.3)	12	(19.2)
Lost of appetite	12	(18.7)	13	(20.9)
Nausea	7	(10.9)	16	(25.8)
Flatulence	5	(7.8)	4	(6.5)

**Table 2:** Parasitological response and adverse events reported after treatment.

	MBZ		SNZ		Estimated differences	95% IC of difference (*I*_1−*α*_)
	No.	%	No.	%	between MBZ-SNZ	[−1, *P*_*s*_ − *P*_*e*_ + *Z*_1−α_*SE]
Efficacy						
Patients with parasitological cure	*55/64	85.9	56/62	90.3	−4.4%	[−1,0.14]
	**55/62	88.7	56/61	91.8	−3.1%	[−1,0.12]
Safety						
Patients with at least one adverse event	15/64	23.4	20/62	32.3	−8.9%	(−26.0; 8.4)
Type of events adverse reported						
Abdominal pain	12 (18.7)		14 (22.6)			
Nausea	5 (7.8)		4 (6.4)			
Bitter taste	2 (3.1)		15 (24.1)			
Diarrhoea	2 (3.1)		3 (4.8)			
Dizziness	0 (0)		6 (9.6)			

*Intention-to-treat analysis for the no inferiority.

**Per-protocol analysis for the no inferiority.
